# Pain modulates neural responses to reward in the medial prefrontal cortex

**DOI:** 10.1002/hbm.24882

**Published:** 2019-11-29

**Authors:** Chenbo Wang, Chaofei Bao, Jiatao Gao, Yujin Gu, Xiao‐Wei Dong

**Affiliations:** ^1^ Key Laboratory of Brain Functional Genomics (MOE&STCSM) Shanghai Changning‐ECNU Mental Health Center, Institute of Cognitive Neuroscience, School of Psychology and Cognitive Science, East China Normal University Shanghai China; ^2^ NYU‐ECNU Institute of Brain and Cognitive Science, New York University Shanghai Shanghai China

**Keywords:** fMRI, mPFC, NAcc, pain, reward

## Abstract

Pain has been found to promote reward‐seeking behaviors, which might be a consequence of modulated brain activities in the reward neural circuitry in a painful state. The present study investigated how pain affected reward processing and reward‐related neural activities using fMRI technique. A total of 50 healthy participants were recruited and used for data analyses, with half being treated with topical capsaicin cream and the other half with hand cream (treatment: pain or control). The participants were asked to perform a card‐guessing game when their brain activities responding to feedbacks (outcome: win or loss) were recorded. Behavioral results showed that participants in pain group overestimated their correct choices in the card‐guess game. Whole‐brain fMRI analysis revealed that the main effect of outcome (win vs. loss) activated a typical network of the reward neural circuitry, including the medial prefrontal cortex (mPFC) and the bilateral nucleus accumbens (NAcc). Importantly, the region of interest analysis revealed a significant interaction of treatment and outcome in the mPFC, with increased mPFC neural activity responding to win outcome in pain condition. Moreover, the functional connectivity between the mPFC and the NAcc was decreased in pain condition. We conclude that the pain‐induced modulation of the mPFC activity could result in alterations of both the emotional response to and the cognitive evaluation of reward.

## 1 INTRODUCTION

Pain‐evoked behaviors are pivotal for human survival. On the one hand, noxious events alert individuals with actual or potential threat and drive them away from harmful situation (Hadjistavropoulos & Craig, 2004). On the other hand, individuals are motivated to fight against a painful situation by accumulating materials or social resources. For example, a group of strangers experiencing painful events together increased their cooperative behaviors and perceived bonding between each other, which may reflect a need for social support (Bastian, Jetten, & Ferris, 2014). Our previous study found that the individuals in acute physical pain displayed more trusting behavior toward their game partners in an attempt to gain more money in an economic decision task (Wang, Gao, Ma, Zhu, & Dong, 2018). Furthermore, the individuals who were motivated to cooperate with others showed enhanced interpersonal neural synchronization in a painful state (Wang et al., 2019). Thus, it is evident that a set of motivated/reward‐seeking behaviors is influenced by pain events. The underlying neural mechanism might involve pain‐induced modulations of brain activities, as it has been well documented that the brain structures and functions that are altered by pain (Apkarian, Bushnell, Treede, & Zubieta, 2005; Rainville, 2002).

The promoted reward‐seeking behavior might be a consequence of modulated neural activities in the reward circuitry in a painful state. First, the reward/motivational neural circuitry could be easily modulated by the pain, as it is engaged in several pain related experiences, including the aversiveness of pain and the reward processing from relief of pain (Navratilova et al., 2012; Navratilova & Porreca, 2014). Second, the modulated neural activities in the reward circuitry are found to be associated with reward‐related behaviors, such as the processing of rewarding music (Seminowicz et al., 2019) and the analgesic effect of placebo (Yu et al., 2014). In detail, brain areas in the reward neural circuitry are known to include the nucleus accumbens (NAcc) in the ventral striatum, the medial prefrontal cortex (mPFC), the orbital prefrontal cortex, and the anterior cingulate cortex (Dillon et al., 2008; Haber & Knutson, 2010; Huckins et al., 2019; Izuma, Saito, & Sadato, 2008). Studies with experimental acute pain found that, both the mPFC activity and its functional connectivity with NAcc were enhanced when subjective pain intensity was self‐regulated (Woo, Roy, Buhle, & Wager, 2015). For chronic pain, such as in patients with fibromyalgia, disrupted brain responses to reward/punishment were observed, with a potentially decreased anticipation of pain relief (Loggia et al., 2014).

In the current study, we aimed to assess how acute pain would modulate brain activities in the reward neural circuitry. Previous reports mainly addressed how reward neural activities contributed to the pain experience, such as pain relief or decreased pain intensity. In our earlier study, however in turn, we found that the increased reward‐seeking behavior in a painful state was associated with an increased anticipation or an overvaluation of the reward (Wang et al., 2018). Thus, the present work is focused to explore how pain altered reward processing, such as the cognitive evaluation and emotional response to reward stimuli. Thus, the mPFC is of particular interest as it plays important roles in the value processing (Gläscher, Hampton, & O'doherty, 2008; Rushworth, Noonan, Boorman, Walton, & Behrens, 2011), anticipated probability (Knutson, Taylor, Kaufman, Peterson, & Glover, 2005), and social contextual (Izuma et al., 2008) aspects of reward processing. We hypothesized that the modulation of the mPFC activity in a painful state was associated with value representation of a reward.

To achieve the goal, we recruited a cohort of 50 participants. Physical pain was induced in half of the participants by topical application of capsaicin cream to one of the forearms in our experiments. The participants were then asked to play a card‐guessing game (Delgado, Nystrom, Fissell, Noll, & Fiez, 2000; Varnum, Shi, Chen, Qiu, & Han, 2014) while their brain activities responding to monetary incentives were recorded by functional magnetic resonance imaging (fMRI) technique. We then measured how pain modulated the mPFC and NAcc neural activities related to reward processing. In addition, we also tested how the functional connectivity between the mPFC and the bilateral NAcc might be changed by pain, as these brain regions in the reward neural circuitry were found interconnected and the connectivity was manipulated by some negative states, such as depression (Felger et al., 2016) and chronic pain (Baliki et al., 2012).

## 2 MATERIALS AND METHODS

### 2.1 Participants

This study first recruited 60 healthy Chinese college students as paid volunteers. Then, a total of 50 participants were used for formal data analyses (25 in pain condition, the other 25 in control condition; 29 females, 21 males; age: 22.4 ± 2.5 years). The age and gender were matched between the two groups. Five participants were excluded due to excessive head motion during fMRI scanning and another five participants due to failure of pain manipulation. The motion criteria for exclusion were the translation of any direction exceeding 4 mm or the rotation of any direction exceeding 3°. The criteria of failure of pain manipulation was that participants rated pain intensity as lower than 3 in pain condition (two participants) or higher than 3 in control condition (three participants). All participants self‐reported no psychiatric illness or chronic pain disorders. This study complied with all provisions of the Declaration of Helsinki and was approved by the University Committee on Human Research Protection of East China Normal University. Written informed consent was obtained prior to participation.

### 2.2 Pain induction and assessment

The procedure of pain induction was the same as what we reported in our previous study (Wang et al., 2018). It was a safe and noninvasive paradigm based on the heat/capsaicin sensitization model (Modir & Wallace, 2010). In the painful treatment, 0.1 mL of Capzasin‐HP cream (capsaicin 0.1%) was brushed to a 2 × 2 cm^2^ area on the volar side of the dominant forearm. And then, the area was covered with plastic film for two reasons: first, to insure skin contact and prevent evaporation; second, similar to a thermode, to accumulate body heat to produce heat allodynia. In the nonpainful treatment, same volume of hand cream was administrated to the same 2 × 2 cm^2^ area. Pain sensation was assessed using a subjective numerical pain rating with an 11‐point visual analog scale (VAS, 0 corresponding to “no pain at all” and 10 corresponding to the “worst imaginable pain”; Carlsson, 1983; Huang et al., 2013).

Pain manipulation check was performed. Pain ratings (Table 1) were assessed with an analysis of variance (ANOVA) of treatment (pain, control) and Time (prescan, during scan, postscan). It revealed a significant main effect of treatment (*F*[1, 48] = 370, *p* < .001). The averaged pain rating during scan was significantly higher in the pain condition than in the control condition (7.09 vs. 0.40, *t*[48] = 20.8, *p* < .001). It demonstrated a successful pain induction that individuals applied with Capzasin experienced sustained moderate pain. Moreover, For the capsaicin group, the pain ratings were not significantly different between each of the two intervals (inter1 = 7.20, inter2 = 7.16, inter3 = 6.88; |*t*s[24]| = 0.57–1.45, *p*s > .15). Thus, the pain effect on brain activities we reported was not likely to be disturbed by the fluctuation of pain intensity.

**Table 1:** Subjective pain assessment in pain and control conditions

	Prescan		During scan			Postscan
	0 min	25 min	Inter 1	Inter 2	Inter 3	60 min
Hand cream	0.40 (0.58)	0.60 (1.3)	0.28 (0.61)	0.32 (0.75)	0.40 (0.76)	0.20 (0.41)
Capsaicin	0.40 (0.65)	5.92 (2.3)	7.20 (1.5)	7.16 (1.7)	6.88 (2.0)	5.60 (2.7)

*Note*: Inter 1 represents the time after run1 (but before run2); similarly, inter 2 is for time after run2, and inter 3 is for time after run3.

### 2.3 Stimuli and procedure

In the experiment, participants were first asked to fill out a few questionnaires, and then either capsaicin cream or hand cream was applied to them. The instruction was identical to all the participants in the two groups. It was as follows: “you will receive either capsaicin cream or hand cream at a chance level and will be aware of that by yourself when it takes effect gradually.” Thirty minutes after pain induction when the capsaicin would produce stable moderate painful feelings, the participant was instructed to lie in the fMRI scanner and play a card‐guessing game. Participants were told that they would have a chance to win extra monetary reward according to their performance in the game in addition to their basic payment (CNY ¥50, ≈ USD $7.50).

**Figure 1:**
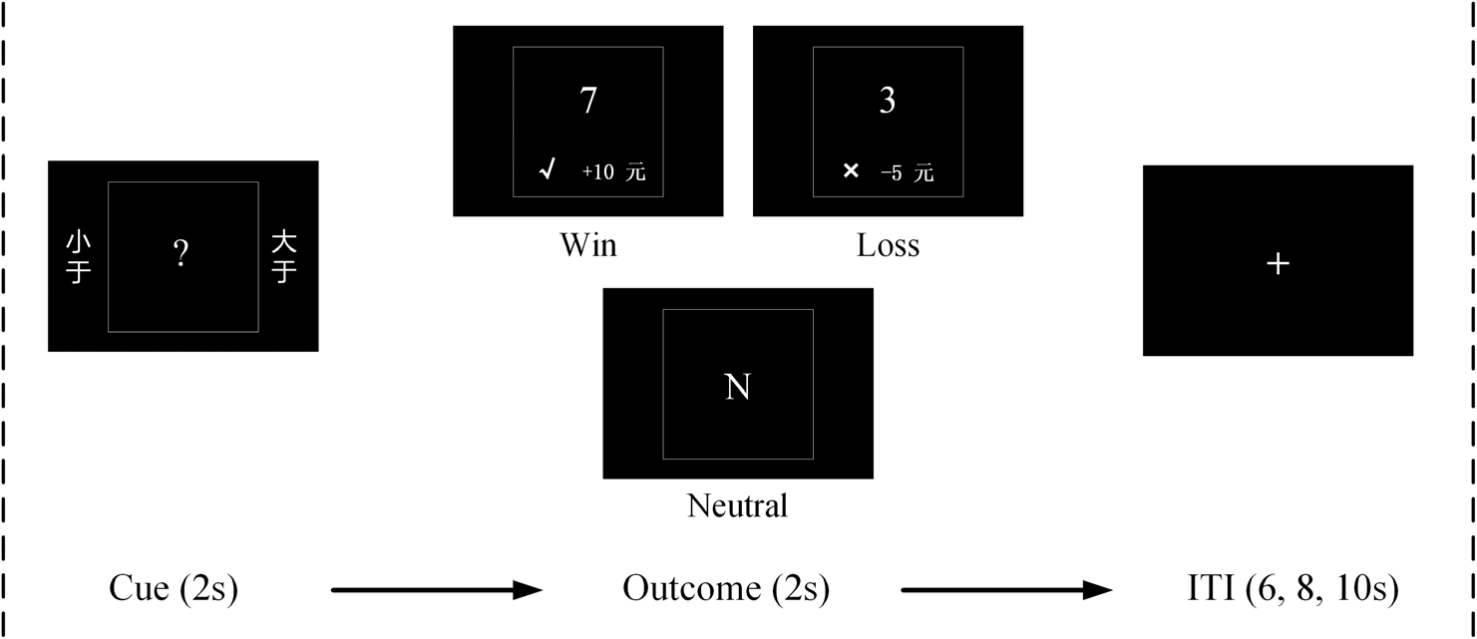
The structure of a trial. Each trial was consisted of a cue, an outcome and an intertrial interval (ITI). The average length of a trial was 12 s. Regarding to the outcome, three types of trials were distinguished: the win, loss, and neutral trials. The procedure was displayed in Chinese. The meaning of these Chinese characters are as follows: “小于” (smaller than), “大于” (larger than)

The card‐guessing game (Delgado et al., 2000) was employed to track brain activations responding to monetary incentives. As illustrated in Figure 1, each trial of this game began with a 2‐second presentation of a card with a “?,” during which the participant guessed whether the number on the card would be smaller or larger than 5 by button press. It was followed by a 2‐second display of an outcome, with one of the following numbers: “1,” “2,” “3,” “4,” “6,” “7,” “8,” and “9.” A “√” was also presented if the participant made a correct guess or a “×” if it was an incorrect guess. A correct guess resulted in a monetary reward of CNY ¥10 (≈USD $1.50) and an incorrect one resulted in a loss of CNY ¥5 (≈USD $0.75; outcome: win/loss). Neutral trial not linked to monetary outcome was included to serve as a baseline, in which a letter “N” was presented. After the outcome, a fixation cross was presented for 6, 8, or 10 s (8 s on average) before the next trial. The average length of a trial was 12 s.

The study involved four functional runs of the card‐guessing task. Each run contained 15 randomly ordered trials which comprised of three types defined by the outcome of the game, including win, loss and neutral (Figure 1). Unbeknownst to participants, the game was programmed so that there would be an equal number of win and loss trials. That was, regardless of the neutral trials, participants would have 50% of correct choices and 50% of incorrect choices. Due to the fixed response accuracy, we thus did not record button presses of their choices. The task took 12 min. After this task, participants were asked to perform another task during which they played the same card‐guessing game for a familiar other but not for themselves. The purpose of this subsequent task was to investigate the influence of pain on vicarious reward, which was not reported in this article. In total, the participants spent 25 min in the scanner. At the end of the experiment, they were randomly paid CNY ¥70~80 (≈USD $10.50–12.00) and debriefed.

#### 2.3.1 Questionnaires

After the completion of all the tasks, participants came out of the scanner; and then, they were asked to evaluate their performance by estimating the percentage of correct choices. They also completed two 7‐point Likert scales (1: very unhappy; 4: neutral; 7: very happy) to indicate how happy they felt when they won/lost the card‐guessing game. Participants assessed pain intensity at each beginning of the four fMRI runs and at the end of scanning.

### 2.4 fMRI acquisition and preprocessing

Image acquisition was performed on a 3.0 T Siemens scanner with a standard head coil of 20 channels at the Shanghai Key Laboratory of Magnetic Resonance of East China Normal University. Functional images were acquired by using T2‐weighted echo‐planar images (EPI) sensitive to blood oxygenation level dependent (BOLD) signal with the following parameters: 64 × 64 matrix, 32 slices, 3.5 × 3.5 × 4.0 mm^3^ spatial resolution, field of view (FOV) = 224 × 224 mm^2^, repetition time (TR) = 2000 ms, echo time (TE) = 30 ms, flip angle (FA) = 90°. High‐resolution anatomical T1‐weighted images were acquired for each participant with the following parameters: 320 × 320 matrix, 40 slices, 0.69 × 0.69 × 3.75 mm^3^ spatial resolution, FOV = 224 × 224 mm^2^, TR = 440 ms, TE = 2.46 ms, FA = 90°.

Functional images were preprocessed using SPM12 (the Wellcome Trust Centre for Neuroimaging, London, UK). The functional data were first adjusted for slice timing to compensate for delays associated with acquisition time differences between slices during the sequential imaging. The images were realigned to the first scan to correct for head motion, normalized into a 3 × 3 × 3 mm^3^ Montreal Neurological Institute (MNI) space, and then spatially smoothed by Gaussian kernel with an isotropic of 8 mm full‐width at half‐maximum (FWHM).

### 2.5 fMRI data analysis

#### 2.5.1 General liner model analysis

General liner model (GLM) was applied to the fMRI data to estimate the effects of different experimental conditions on brain activations. For the first‐level analysis, brain images recorded during the 2‐second outcome trials were modeled in the GLM, where parameter estimations were conducted by convolving regressors with canonical hemodynamic response function (HRF). Using the neutral condition as a baseline of metabolism, three regressors win, loss, and neutral trials were set to [1, 0, −1] accounting for the win versus neutral contrast, and [0, 1, −1] for the loss versus neutral contrast. Meanwhile, six motion parameters (translation: *x*, *y*, *z*; rotation: pitch, roll, yaw) were also included in the model to account for effects of no interest. For the second‐level analysis, the first‐level contrast images were subjected to an ANOVA of outcome (win, loss) and treatment (pain, control). This whole‐brain analysis was expected to identify brain activations that were modulated by reward and loss outcomes, by acute experimental pain, or by the interaction of these two settings. Significant brain activations were determined by a cluster‐level threshold of *p* < .05, FWE corrected. In addition, post hoc whole‐brain analyses of win versus neutral and loss versus neutral contrasts were conducted separately for each group, with an exploratory threshold of *p* < .001, uncorrected, cluster size *k* > 50.

#### 2.5.2 Region of interest analysis

Region of interest (ROI) analysis was conducted to examine the effect of pain on reward neural activity. Three ROIs related to reward processing were selected based on the main effect of outcome in this study, which exhibited greater activations in the win condition compared to the loss condition: the mPFC (−3/41/−5), left NAcc (left NAcc, −15/5/−8), and right NAcc (15/5/−11). These ROIs were defined as spheres with a radius of 6 mm centered at those MNI co‐ordinates using MarsBaR (http://marsbar.sourceforge.net). Beta values of win condition and loss condition were extracted in contrast with neutral condition. The contrast values of (win > neutral) and (loss > neutral) were then subjected to a repeated‐measures ANOVA with outcome (win, loss) as a within‐subject variable and treatment (pain, control) as a between‐subject variable.

#### 2.5.3 Psychophysiological interactions analysis

Generally, psychophysiological interaction (PPI) approach was developed to examine how the functional connectivity between regions changed with experimental condition (Friston et al., 1997). We performed PPI analysis to investigate which brain areas showed activations co‐varying with mPFC activity when pain was applied. BOLD time series during the 2‐second outcome stage of each trial were extracted from a sphere with a radius of 6 mm centered at the maximum of the win > loss contrast over the MPFC. Three regressors of a design matrix were defined for PPI analysis: the win versus loss contrast as a psychological variable, the MPFC neural activation as a physiological variable, and the interaction of these two. As we were particularly interested in the context dependent contribution of the mPFC (a seed region) to other reward‐related brain areas (regions‐of‐interest), PPI estimates reflecting the strength of functional connectivity of predefined pairs of brain regions (i.e., MPFC—left NAcc, MPFC—right NAcc) were thus extracted and compared between pain and control groups.

## 3 RESULTS

### 3.1 Behavioral results: A positive bias of performance estimation in pain

**Figure 2:**
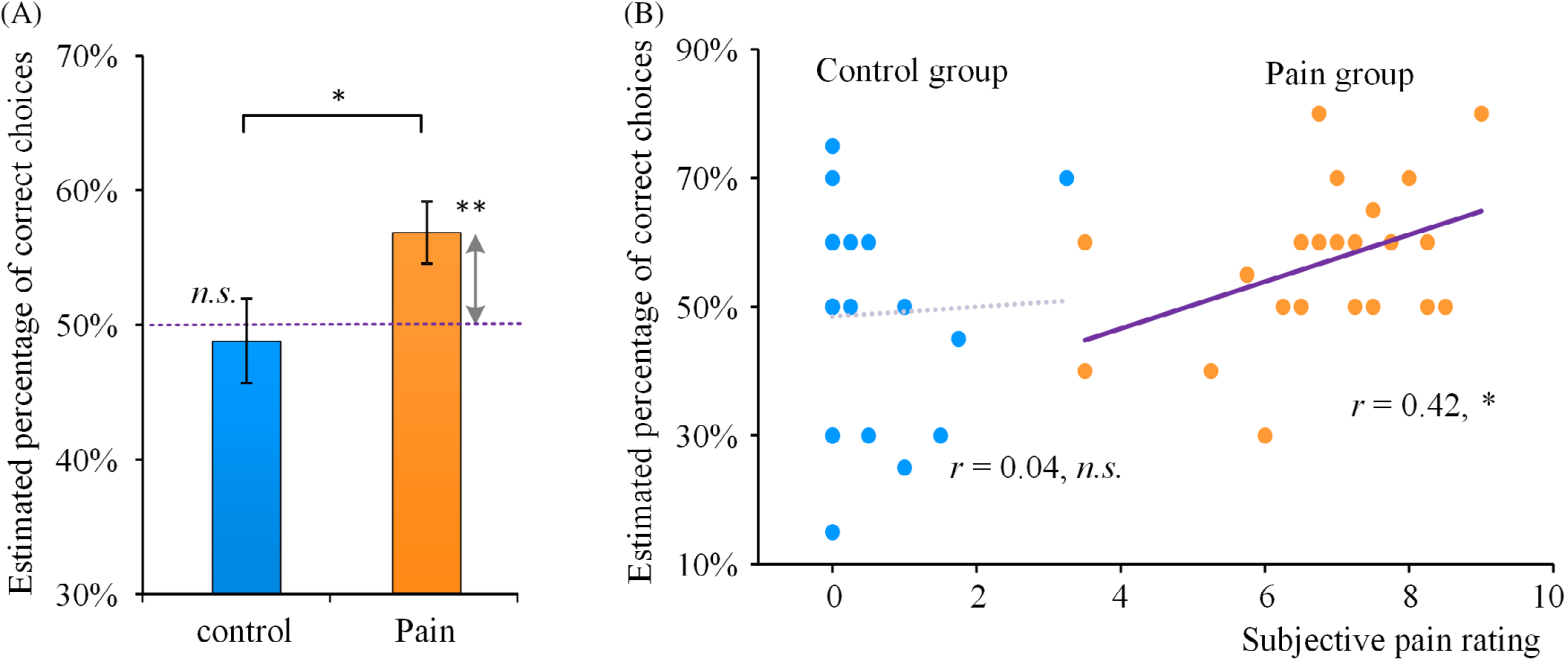
A positive bias of performance estimation in pain. (a) Estimated percentages of correct choices in the pain and control conditions were compared to the actual accuracy of 50%. (b) Correlations between estimated percentage of correct choices and subjective pain ratings were displayed respectively for the pain group and the control group. The *, **, and *n.s*. denote *p* < .05, *p* < .01, and not significant, respectively

To assess the effect of acute pain on reward‐related behavior, participants were asked to estimate the percentage of correct choices they had made in the card‐guessing game. Participants in pain group believed they made 56.8% correct choices, which was significantly higher than the actual accuracy (compared to 50%, *t*[24] = 2.95, *p* = .007; Figure 2a); whereas participants in control group reported an accurate estimation of their performance (48.8%, *p* > .5). The estimation was significantly different between the two groups (*t*[48] = 2.05, *p* = .046). It suggested that individuals experiencing pain exhibited a positive bias of performance estimation. Furthermore, the estimation of correct choices was positively correlated with subjective pain rating in the pain group (*r* = .42, *p* = .037, Figure 2b) but not in the control group (*r* = .04, *p* = .862), suggesting that individuals who felt more painful tended to perceive more beneficial outcome.

### 3.2 GLM analysis: Reward‐related whole‐brain activation

**Figure 3:**
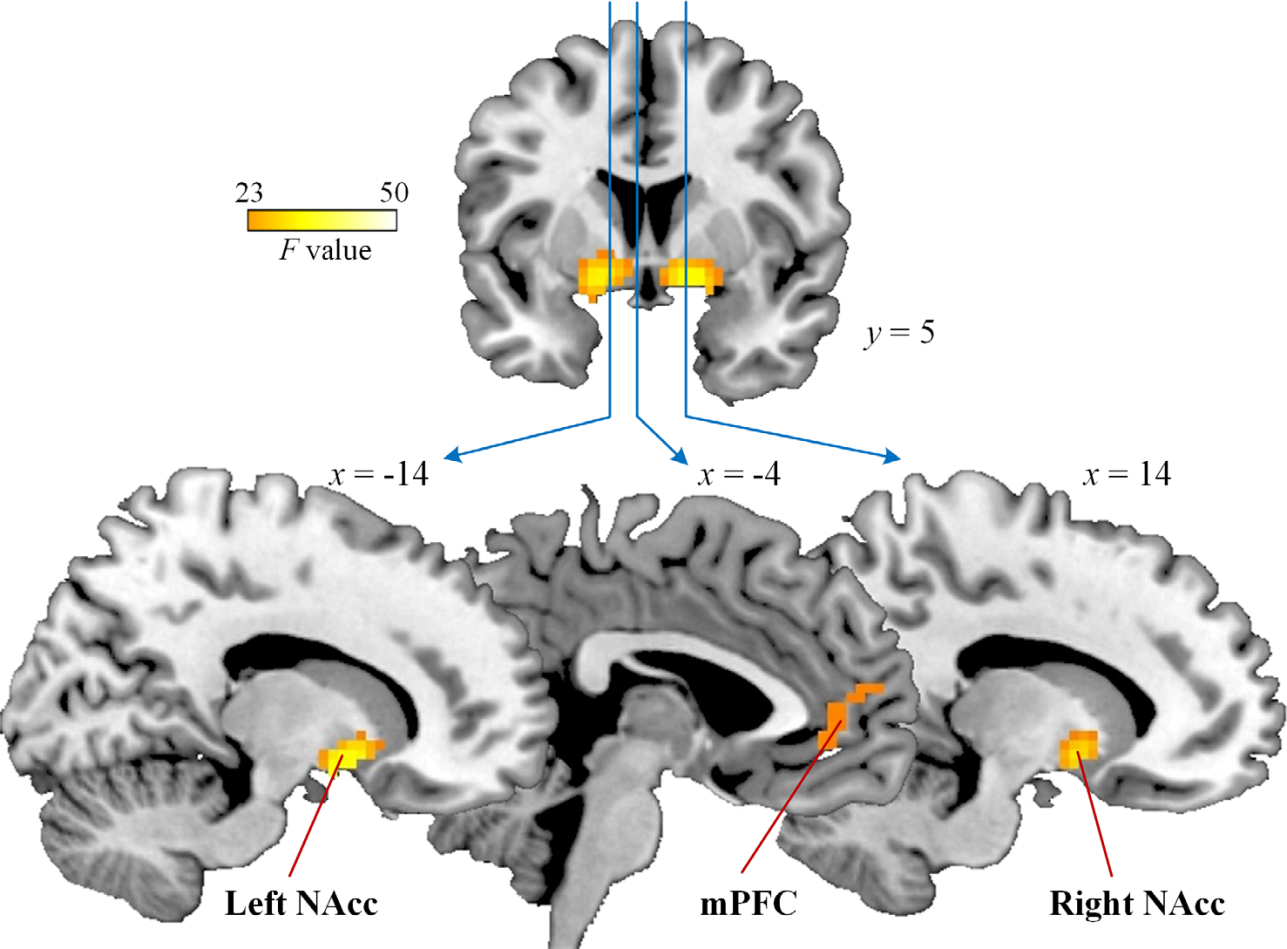
Neural activation in whole‐brain analysis of the main effect of outcome. The threshold was *p* < .05, FWE corrected at the cluster‐level. Color bar denotes *F* value of the contrast. mPFC, medial prefrontal cortex; NAcc, nucleus accumbens

Whole‐brain analysis, with a cluster‐level threshold of *p* < .05 FWE corrected, was conducted by an ANOVA of outcome (win, loss) and treatment (pain, control). The main effect of outcome revealed significant brain activation at regions including the mPFC (−3/41/−5), the left NAcc (−15/5/−8) and right NAcc (15/5/−11, Table 2 and Figure 3). These activations constituted a typical network of the reward neural circuitry (Varnum et al., 2014). However, the main effect of treatment revealed no significant brain activations. Neither did the interaction between treatment and outcome.

**Table 2:** Whole‐brain activation regarding the main effect of feedback

Brain region	*k* (voxels)	*F* value	Peak co‐ordinates		
			*x*	*y*	*z*
Right nucleus accumbens	93	61.80	15	5	−11
Left nucleus accumbens	78	47.86	−15	5	−8
Medial prefrontal cortex	11	26.21	−3	41	−5
Medial prefrontal cortex	5	25.31	−6	56	13
Medial prefrontal cortex	2	26.23	−6	41	19
Left superior frontal gyrus	3	25.87	−18	32	55

*Note*: Threshold: *p* < .05, FWE corrected at the cluster‐level.

Post hoc whole‐brain analysis confirmed that the brain activations regarding the main effect of outcome were dominantly contributed by the win condition, but not by the loss condition, using the neutral condition as a baseline. First, for whole‐brain analysis of win versus neutral contrast, the mPFC, the bilateral NAcc and other brain regions were activated in both the control group (Table S1 and Figure S1) and the pain group (Table S2 and Figure S1), with an exploratory threshold (*p* < .001, uncorrected, *k* > 50). However, no significant difference was found between groups. Second, for whole‐brain analysis of loss versus neutral contrast, no brain regions were identified in both the control group and the pain group. Greater activations over the bilateral insula and frontal gyrus that were found to encode monetary loss in previous study (Varnum et al., 2014) were only observed with a much looser but unaccepted threshold (*p* < .01, uncorrected).

### 3.3 ROI analysis: Effects of pain on reward‐related brain activation

**Figure 4:**
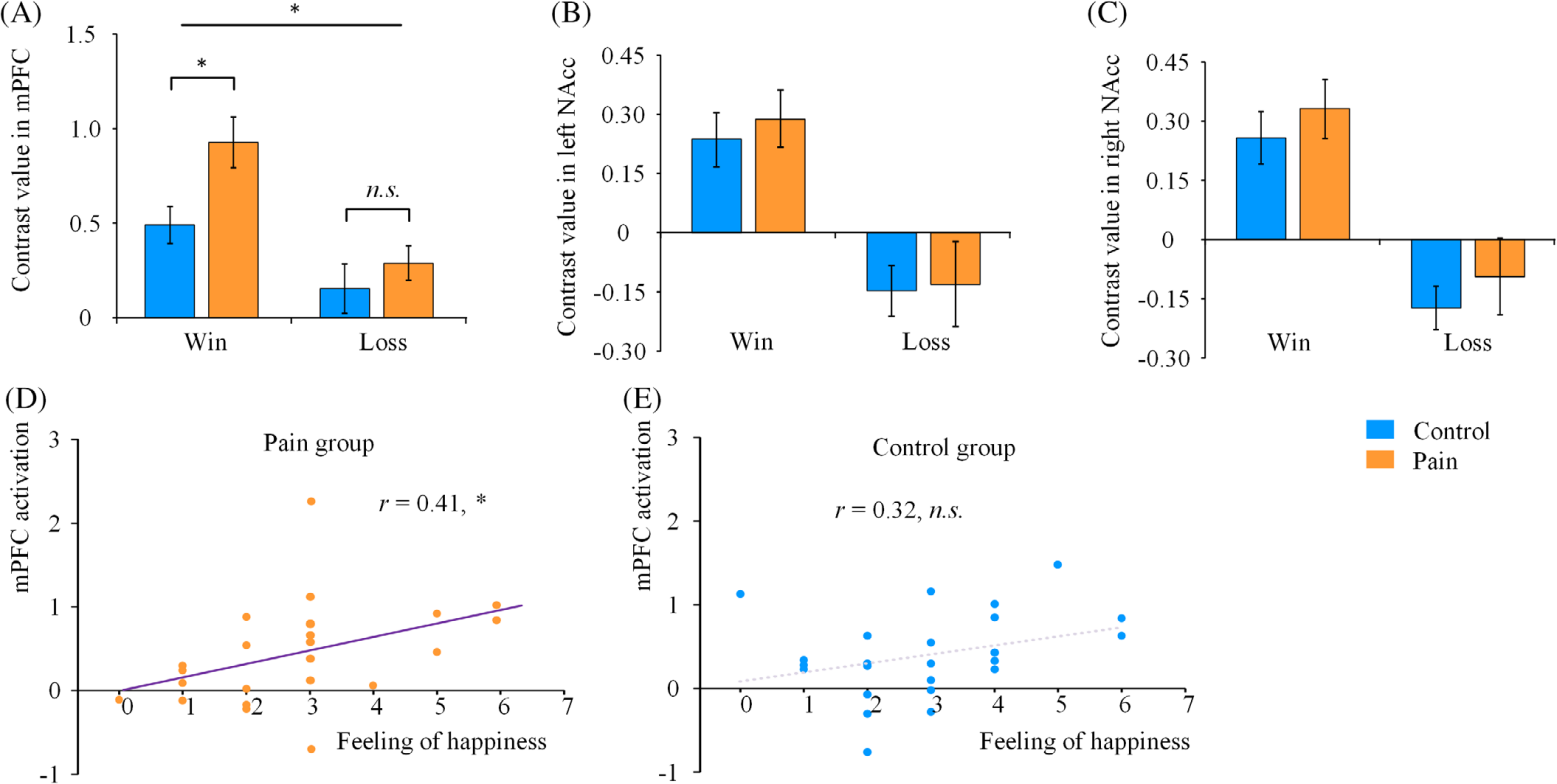
Effects of pain on reward‐related brain activation. Contrast values of win versus neutral, loss versus neutral condition were extracted, respectively, from the pain and control groups in three predefined ROIs including the mPFC (a), left NAcc (b), and right NAcc (c). Error bars denote *SE*. Correlations between the mPFC activation of the win versus loss contrast and subjective rating of happy feeling (win vs. loss) were displayed, respectively, for the pain group (d) and the control group (e). The * and *n.s*. denote *p* < .05 and not significant, respectively

ROI analysis was conducted to examine the effect of pain on reward‐related neural activity. For each of three ROIs including the mPFC (−3/41/−5), the left NAcc (−15/5/−8) and the right NAcc (15/5/−11), contrast values were subjected to an ANOVA of outcome (win, loss) and treatment (pain, control). It revealed significant main effects of outcome in all three regions (*F*s[1, 48] = 41.4–49.1, *p*s < .001; Figure 4), suggesting stronger neural activities responding to monetary win than to monetary loss in the reward neural circuitry. For the main effect of treatment, a marginally significant result was found in the mPFC (*F*[1, 48] = 3.68, *p* = .061), but not in the bilateral NAcc (*F*s[1, 48] < 1, *p*s > .3). It suggested stronger mPFC activation potentially in the pain group compared to the control group.

Of most interest, a significant interaction of outcome * treatment was observed in the mPFC (*F*[1, 48] = 4.75, *p* = .034). When separated by outcomes, independent sample *t* tests showed that pain enhanced mPFC activity responding to monetary win feedback (*t*[48] = 2.63, *p* = .012), but not to monetary loss feedback (*t*[48] = .83, *p* = .414; Figure 4a). The findings indicate that acute experimental pain modulates neural activities to the monetary reward stimuli. In addition, no significant interaction effect was found in the bilateral NAcc (*F*s[1, 48] < 1, *p*s > .5). These results suggest that only the mPFC in the reward neural circuitry is subjected to the modulation of pain on reward processing.

Emotional response to monetary win and loss was measured by the degree of self‐report happy feeling (see Table 3). Correlation analysis showed that the mPFC activity (win vs. loss) was positively correlated with the degree of happy feeling (win vs. loss) in the pain group (*r* = .44, *p* = .029; Figure 4d). The correlation was not significant in the control group (*r* = .19, *p* = .363; Figure 4e). It suggested that neural response in the mPFC was associated with a positive emotional response to monetary reward in a painful situation.

**Table 3:** Subjective rating of happy feeling to the win and loss feedback

Treatment	Win feedback	Loss feedback	Differences (win–loss)
Control group	5.80 (0.96)	2.84 (0.99)	2.96 (1.51)
Pain group	5.76 (0.83)	2.88 (0.88)	2.88 (1.59)

### 3.4 PPI analysis: Effects of pain on reward‐related functional connectivity

**Figure 5:**
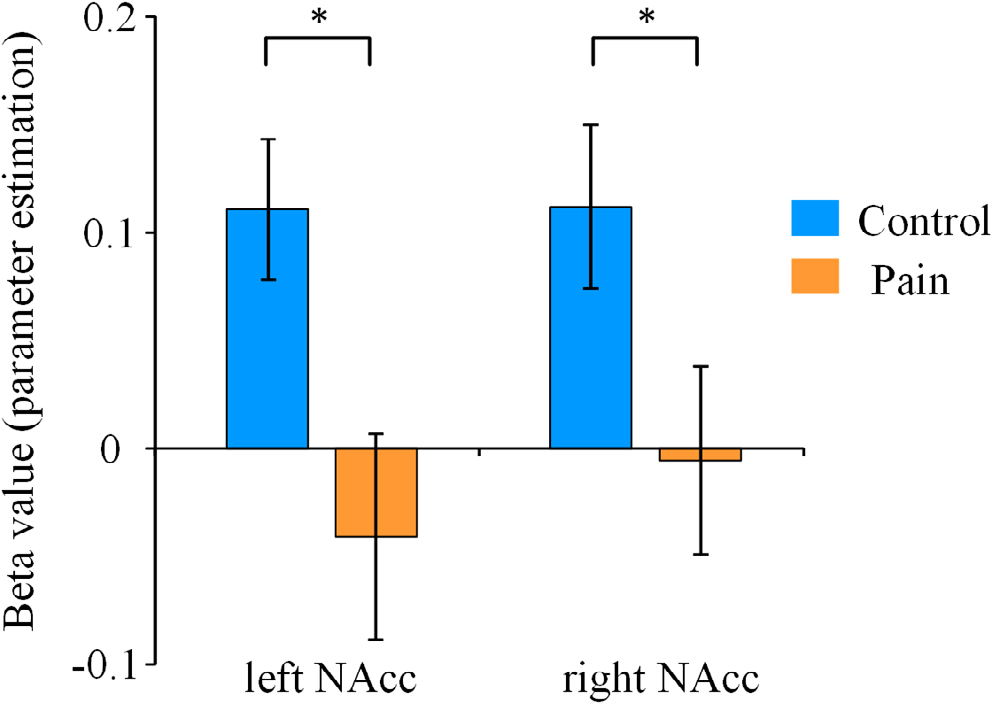
Task‐dependent functional connectivity between the mPFC and bilateral NAcc. Beta values of task‐dependent couplings between the mPFC and the bilateral NAcc were extracted in PPI analysis. Error bars denote *SE*. The * denotes *p* < .05

Psychophysiological interactions (PPI) analysis was performed to examine how acute pain may change the functional connectivity between reward‐related brain regions, as they were usually interconnected with each other. Beta values of task‐dependent couplings between the seed region (i.e., mPFC) and the regions‐of‐interest (i.e., the left and right NAcc) were extracted. Independent sample *t* tests revealed that the beta values in bilateral NAcc in the pain group were much lower than those in the control group (left: *t* (36) = −2.64, *p* = .012; right: *t* (36) = −2.04, *p* = .049, Figure 5). The results indicated that pain might eliminate the task‐dependent (win > loss) couplings between the mPFC and the bilateral NAcc which were evident in nonpainful individuals.

## 4 DISCUSSION

The present study investigated the influence of pain on neural responses to reward in the brain regions containing the reward/motivation circuitry. Behavioral results showed that the participants in pain group overestimated their correct choices in the card‐guess game. The fMRI results revealed that the brain areas in the typical reward neural circuitry, including the mPFC and the bilateral NAcc, responded to the reward outcome in both the pain and control groups. Interestingly, ROI analysis found that the mPFC activity showed a significant interaction between treatment and outcome. It was further confirmed that physical pain increased reward‐related neural activity in the mPFC. The mPFC activity was positively correlated with the subjective rating of happy feeling related to monetary gain in pain condition. Moreover, the functional connectivity between the mPFC and the NAcc was decreased in pain condition. These findings demonstrate that the reward‐related activity of the mPFC is subject to the modulation of pain.

The mPFC has been recognized as one of the key brain regions comprising the reward neural circuitry (Dillon et al., 2008; Haber & Knutson, 2010; Huckins et al., 2019; Izuma et al., 2008). It plays an important role in encoding the probability and/or the value of a reward (Gläscher et al., 2008; Hu, 2018; Rushworth et al., 2011; Samejima & Doya, 2007). In this regard, the augmented mPFC activation observed in pain condition might reflect an overvaluation of the reward. Indeed, the mPFC has been found to contribute to reinterpret the meaning of an event (Ochsner & Gross, 2005; Quirk & Beer, 2006). Using the reinterpreted information, the mPFC thus regulates emotional state by suppressing a negative emotion or intensifying a pleasant experience (Etkin, Egner, & Kalisch, 2011; Ochsner & Gross, 2005). This assumption may explain our finding that individuals who exhibited greater reward‐related mPFC activation reported stronger subjective happy feeling, as the reward stimulus being reappraised more valuable. Therefore, our findings demonstrate that the mPFC plays an essential role in pain‐induced modulation of reward processing. Furthermore, it reveals that the enhanced mPFC activity may contribute to a pleasant experience of emotion valuation associated with monetary gains.

Meanwhile, reward‐related bilateral NAcc activities were not influenced by pain. According to a theory of dopamine‐mediated reward prediction‐error signaling proposed by Schultz (2016), there are two components related to the processing of reward, detection and valuation. The NAcc is a pivotal region for processing of the hedonic component of reward (Mano & Seymour, 2015; Smith & Berridge, 2007). Thus, our findings indicate that the detection of reward may not be affected by pain. However, we cannot conclude that the NAcc neural activity is unaffected by pain when it is responsible for other aspects of reward processing, as the NAcc is also found to encode the reward probability during reinforcement learning (Abler, Walter, Erk, Kammerer, & Spitzer, 2006; Knutson, Adams, Fong, & Hommer, 2001). We admit that the win probability in the card‐guessing game were just 50% in our study, in which no reinforcement learning was involved. Therefore, further investigation is needed to determine whether pain alters NAcc neural activities that process reward probability, by adopting a learning task with prediction errors, such as a monetary incentive task (Abler et al., 2006).

In our study, the acute pain decreased the task‐dependent coupling between the bilateral NAcc and the mPFC, indicating a weakened communication between these regions in the reward neural circuitry in pain. This was probably due to a change occurring only at one side of the two interconnected areas, such as an increase of the mPFC activity. Actually, the NAcc receives substantial projections from the prefrontal cortex. This corticolimbic connection is thought to be engaged in emotional appraisal and valuation (Woo et al., 2015), as well as goal‐directed behaviors such as reward seeking (Hu, 2018; Sesack & Grace, 2010). It was also reported by a longitudinal chronic pain study that the NAcc‐mPFC functional connectivity predicted the transition from acute to chronic pain state (Baliki et al., 2012). Therefore, the interrupted corticolimbic connectivity observed in the current study might be responsible for the biased goal‐directed behavior, such as an overestimation of their performance in the card‐guess game in our study. However, this speculation needs to be confirmed by further investigation.

The current work extends our understanding about why pain may promote prosocial behaviors. It was found that pain promoted interpersonal trust (Wang et al., 2018), enhanced cooperative behavior (Bastian et al., 2014), and strengthened interpersonal brain synchronous associated with cooperation (Wang et al., 2019). We assume that an enhancement of reward valuation induced by pain may have impacts on a set of social behaviors. Some prosocial behaviors, such as trusting more on or cooperating more with others can be rewarding in two aspects: first, it may result in accumulating resources by exchanging materials among social members; second, participants may gain social support in an emotion‐sharing way. Thus, by enhancing reward valuation, an acute painful state may increase the likelihood of performing prosocial behaviors. However, this assumption of linking among pain, reward and prosocial behavior awaits to be verified by more direct evidence, as no social association is examined in the current task in the study.

Our observation of the enhancement of both reward‐related behaviors and neural activities in the acute pain condition provides important information for the understanding of the development of chronic pain. Unlike acute pain, patients with chronic pain are frequently associated with depression (Breivik, Collett, Ventafridda, Cohen, & Gallacher, 2006; Gureje et al., 2008), which leads to clinically manifested anhedonia and diminished motivation for natural reinforcers. As a result, chronic pain patients may exhibit social withdrawal behaviors, such as reduced empathy (Peng et al., 2019) and dissatisfaction with social activities (Kerns, Rosenberg, & Otis, 2002). These findings support a combined reward deficiency and antireward model in pain chronification (Borsook et al., 2016). Thus, it appears that the reward system displays opposing responses to acute (enhancement) and chronic (diminution) pain conditions. It is tempting to speculate that the reward system is at first in a hyperactive state with acute pain, but with the continuation of pain, it may then gradually change into an inactive state. Nevertheless, further investigations are needed to measure the longitude changes of the reward neural circuitry among pain patients and then assess how these changes may contribute to the chronification of pain.

It also worth noting some limitations of the current work. First, the relatively small sample size restricted us from conducting further analyses to examine a potential gender effect. By independent sample *t* tests, we found that the mPFC activity responding to the win feedback was relatively higher in the pain condition than that in the control condition in females (*t*[27] = 2.21, *p* = .036), but not in males (*t*[27] = 1.05, *p* = .309). This to some extent may verify the prosocial effect of pain observed predominantly in females (Wang et al., 2018). However, the triple interaction of outcome, treatment and sex was insignificant (*F*[1, 46] < 1, *p* = .78). Thus, to assess this potential gender effect, it warrants further investigation with a decent sample size. Second, the correlation between the mPFC activity and happy feeling was only observed in the win–loss contrast. We conducted a much straightforward correlation analysis between the mPFC activity in win condition and the happy feeling to win feedback. However, this yielded no significant results in both the pain group (*r* = .13, *p* = .537) and the control group (*r* = .13, *p* = .546). It might be due to low discrimination of the rating of happy feeling, as the rating scale was merely from 4 (neutral) to 7 (very happy). Third, we did not measure the unpleasantness of pain, which prevented us from conducting further analyses to assess how the unpleasant feeling of pain may influence the happy feeling of the reward, and vice versa.

In summary, the present study demonstrates a facilitatory effect of acute pain on the activities of reward‐related neural circuitry by showing that pain increased neural responses to monetary incentives in the mPFC. We conclude that the pain‐induced modulation of the mPFC activity could result in alterations of both the emotional response to and the cognitive evaluation of reward. These findings provide important information for the understanding of the impact of acute pain on reward/motivational circuitry as well as the reward‐seeking behavior.

## CONFLICT OF INTEREST

The authors declare no conflicts of interest.

## AUTHOR CONTRIBUTIONS

C.W., C.B., and X.‐W.D. designed the study. C.W., J.G., and Y.G. performed the data collection. C.W., C.B., J.G., and Y.G. analyzed the data. C.W., C.B., and X.‐W.D. wrote the manuscript. All authors read and provided final approval of the article.

## Supporting information


**Table S1** Whole‐brain activation (win vs. neutral, control group)
**Table S2**. Whole‐brain activation (win vs. neutral, pain group)
**Figure S1**. Neural activation in whole‐brain analysis of win versus neutral contrast for each group. Brain activations were found similarly between the control group (a) and the pain group (b). The threshold was voxel level *p* < .001, uncorrected. Color bar denotes t values of the contrast. The mPFC: medial prefrontal cortex; NAcc: nucleus accumbens; MOG: middle occipital gyrus; MCC: middle cingulate cortex.

## Data Availability

Datasets are available on request. The raw data and generated data during analyses supporting the conclusions of this manuscript will be made available by the authors, without undue reservation, to any qualified researcher.
