# Supplementary material for: Pain modulates neural responses to reward in the medial prefrontal cortex
Source: Hum Brain Mapp. 2019 Nov 29;41(5):1372–81. doi: 10.1002/hbm.24882 (PMC7267926; doi:10.1002/hbm.24882)
Supplement: Supplementary file 1 — Table S1 Whole‐brain activation (win vs. neutral, control group) Table S2. Whole‐brain activation (win vs. neutral, pain group) Figure S1. Neural activation in whole‐brain analysis of win versus neutral contrast for each group. Brain activations were found similarly between the control group (a) and the pain group (b). The threshold was voxel level p < .001, uncorrected. Color bar denotes t values of the contrast. The mPFC: medial prefrontal cortex; NAcc: nucleus accumbens; MOG: middle occipital gyrus; MCC: middle cingulate cortex. [file HBM-41-1372-s001.docx]

**Supplementary Materials**

**Table S1. Whole brain activation (Win *vs* Neutral, control group)**

| Brain region | k  (voxels) | t  value | peak coordinates | | |
| --- | --- | --- | --- | --- | --- |
|  |  |  | *x* | *y* | *z* |
| Medial Prefrontal Cortex | 1104 | 6.16 | -6 | 32 | -11 |
| Right Nucleus Accumbens | 72 | 5.87 | 15 | 8 | -8 |
| Left Middle Occipital Gyrus | 930 | 9.05 | -15 | -106 | 4 |
| Middle cingulate cortex | 165 | 5.89 | 3 | -25 | 40 |
| Left Inferior Frontal Gyrus | 64 | 4.92 | -27 | 17 | -20 |
| Right Inferior Frontal Gyrus | 97 | 6.37 | 30 | 17 | -20 |
| left Temporo-parietal Junction | 411 | 6.54 | -51 | -64 | 40 |
| Right Temporo-parietal Junction | 55 | 4.36 | 30 | -61 | 52 |
| Precuneus | 62 | 3.80 | 0 | -55 | 40 |

Threshold: p < 0.001, uncorrected, with cluster size k > 50.

Note: the Left Nucleus Accumbens were activated with a cluster size of 30.

**Table S2. Whole brain activation (Win *vs* Neutral, pain group)**

| Brain region | k  (voxels) | t  value | peak coordinates | | |
| --- | --- | --- | --- | --- | --- |
|  |  |  | *x* | *y* | *z* |
| Medial Prefrontal Cortex | 1262 | 7.09 | 0 | 44 | 16 |
| Left Nucleus Accumbens | 81 | 4.53 | -21 | -7 | -11 |
| Right Nucleus Accumbens | 52 | 4.84 | 15 | 5 | -8 |
| Left Middle Occipital Gyrus | 444 | 8.33 | -12 | -106 | 4 |
| Middle cingulate cortex | 66 | 4.87 | 0 | -16 | 34 |
| Right Middle Frontal Gyrus | 65 | 4.85 | 36 | 44 | -8 |
| Right Temporo-parietal Junction | 60 | 4.82 | -48 | -64 | 37 |
| Posterior Cingulate Cortex | 54 | 3.92 | -3 | -70 | 7 |
| Precuneus | 57 | 4.04 | -3 | -55 | 25 |

Threshold: p < 0.001, uncorrected, with cluster size k > 50.


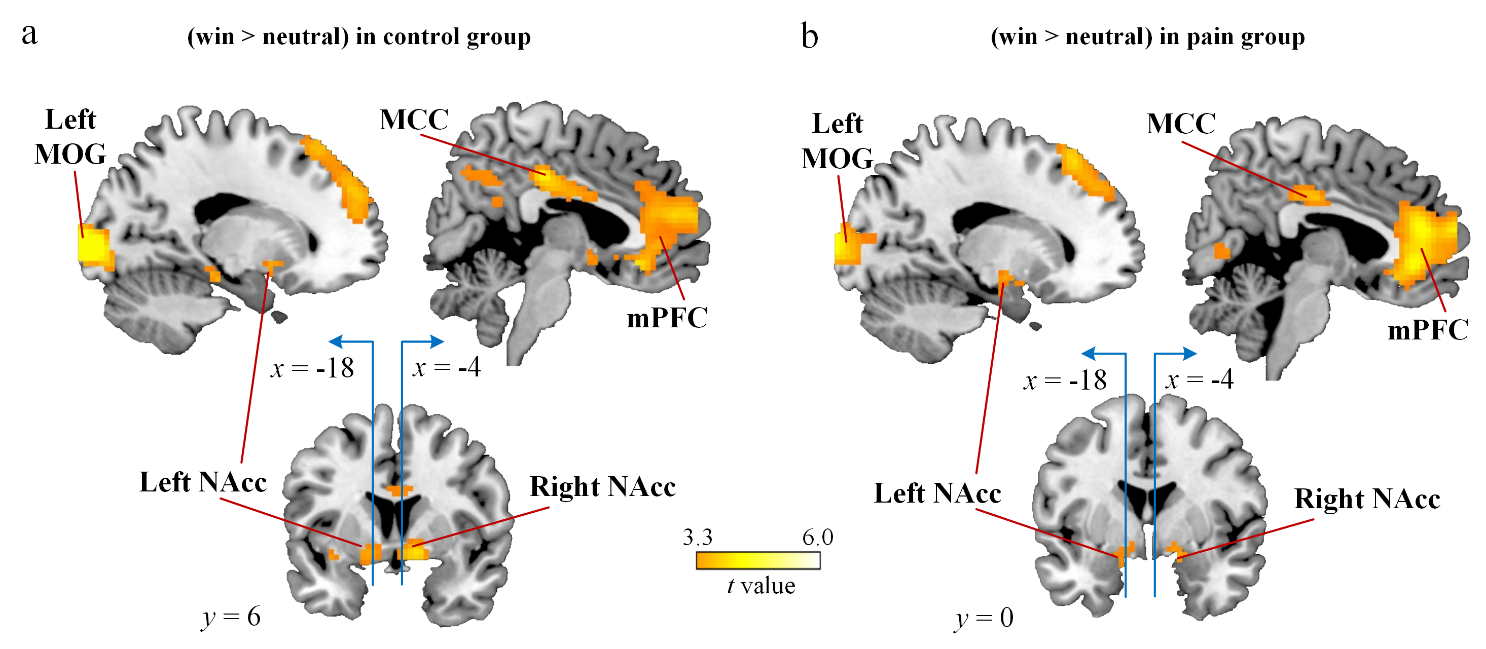


**Figure S1.** **Neural activation in whole brain analysis of Win *vs* Neutral contrast for each group.** Brain activations were found similarly between the control group (a) and the pain group (b). The threshold was voxel level p < 0.001, uncorrected. Color bar denotes t values of the contrast. The mPFC: Medial Prefrontal Cortex; NAcc: Nucleus Accumbens; MOG: Middle Occipital Gyrus; MCC: Middle Cingulate cortex.
